# On discovering functions in actin filament automata

**DOI:** 10.1098/rsos.181198

**Published:** 2019-01-23

**Authors:** Andrew Adamatzky

**Affiliations:** Unconventional Computing Lab, University of the West of England, Bristol, UK

**Keywords:** actin, computing, automata

## Abstract

We simulate an actin filament as an automaton network. Every atom takes two or three states and updates its state, in discrete time, depending on a ratio of its neighbours in some selected state. All atoms/automata simultaneously update their states by the same rule. Two state transition rules are considered. In semi-totalistic Game of Life like actin filament automaton atoms take binary states ‘0’ and ‘1’ and update their states depending on a ratio of neighbours in the state ‘1’. In excitable actin filament automaton atoms take three states: resting, excited and refractory. A resting atom excites if a ratio of its excited neighbours belong to some specified interval; transitions from excited state to refractory state and from refractory state to resting state are unconditional. In computational experiments, we implement mappings of an 8-bit input string to an 8-bit output string via dynamics of perturbation/excitation on actin filament automata. We assign eight domains in an actin filament as I/O ports. To write True to a port, we perturb/excite a certain percentage of the nodes in the domain corresponding to the port. We read outputs at the ports after some time interval. A port is considered to be in a state True if a number of excited nodes in the port's domain exceed a certain threshold. A range of eight-argument Boolean functions is uncovered in a series of computational trials when all possible configurations of eight-elements binary strings were mapped onto excitation outputs of the I/O domains.

## 1. Introduction

Ideas of information processing on a cytoskeleton network have been proposed by Hameroff and Rasmussen in late 1980s in their designs of tubulin microtubules automata [1] and a general framework of cytoskeleton automata as sub-cellular information processing networks [2,3]. Priel, Tuszynski and Cantiello developed a detailed concept on how information processing could be implemented in actin-tubulin networks of neuron dendrites [4]. A signal transmission along the microtubules is implemented via travelling localized patterns of conformation changes or orientations of dipole moments of the tubulin units in tubulin microtubules and ionic waves in actin filaments. A high likelihood of existence of travelling localizations (defects, ionic waves and solitons) in tubulin microtubules and actin filaments is supported by a range of independent (bio)-physical models [5–12]. A convincing hypothesis is that actin networks in synaptic formations play a role of filtering/processing input information which is further conveyed to and amplified by tubulin microtubules. Thus, in the present paper we focus on actin filaments.

Actin is a protein presented in all eukaryotic cells in forms of globular actin (G-actin) and filamentous actin (F-actin) [13–15]. G-actin polymerizes in double helix of filamentous actin; during polymerization G-actin units slightly change their shapes and thus become F-actin units [16]. The actin networks play a key role in information processing [17–20] in living cells. Previously, we have demonstrated how to implement Boolean, multi-valued and quantum logical gates on coarse-grained models of actin filaments using cellular automata, quantum automata and a lattice with Morse potential approaches [21–25]. Theoretical designs of actin-based logical circuits realize logical gates via collisions between travelling localizations. Such an approach assumes that we can address nearly every atom in the actin molecule [26] or control exact timing of the collisions between travelling localizations [25]. Such assumptions might prove to be unrealistic when experimental laboratory implementations are concerned. This is why, in the present paper, we consider a less restrictive, than in previous implementations, way of executing computation on protein polymer: to probe relatively large portions of an actin filament as I/O and uncover Boolean functions implemented via input to output mapping. The approach proposed is novel and has not been considered before. Another original feature of the presented results is that we employ a detailed model of several actin units arranged in the helix. The model is introduced in §2. To discover Boolean functions implementable in the actin filament, we split the helix into eight domains. We perturb the domains in all possible combinations of excitation representing the state of 8-bit strings and record their outputs. A mapping between an input and output sets of binary strings is constructed then. This is shown in §3. We discuss limitations of the approach and future developments in §4.

## 2. Actin filament automata

**Figure 1.**
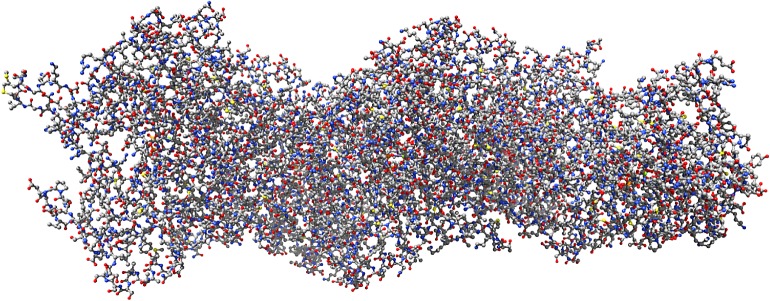
A pseudo-atomic model of F-actin [27] in Corey–Pauling–Kolun colouring.

We employed a pseudo-atomic model of an F-actin filament (figure 1) reconstructed by Galkin *et al*. [27] at 4.7 Å resolution using a direct electron detector, cryoelectron microscopy and the forces imposed on actin filaments in thin films.^1^ The model has 14 800 atoms and is composed of six F-actin molecules. Following our previous convention [28], we represent an F-actin filament as a graph F=⟨V, E, C, Q, f⟩, where **V** is a set of nodes, **E** is a set of edges, **C** is a set of Euclidean coordinates of nodes from **V**, **Q** is a set of node states, f : Q×[0,1]→Q is a node state transition function, calculating next state of a node depending on its current state and a ratio of excited neighbours belonging to a sub-interval of [0, 1]. Each atom from a pseudo-atomic model of an F-actin filament is represented by a node from **V** with its three-dimensional coordinates being a member of **C**; atomic bonds are represented by **E**. Each node *p* ∈ **V** takes states from a finite set **Q**. All nodes update their states simultaneously in discrete time. A node *p* updates its state depending on its current state *p*^*t*^ and ratio *γ*(*p*)^*t*^ of its neighbours being in some selected state ⋆. We consider two types of a node neighbour. Let *u*(*p*) be nodes from **V** that are connected with an edge with a node *p*; they correspond to atoms connected by the chemical bonds with atom *p*. We call them hard neighbours because their neighbourhood is determined by the chemical structure of F-actin. The ratio of nodes with one hard neighbour is 0.298, two hard neighbours 0.360, three hard neighbours 0.341 and four hard neighbours 0.001.

**Figure 2.**
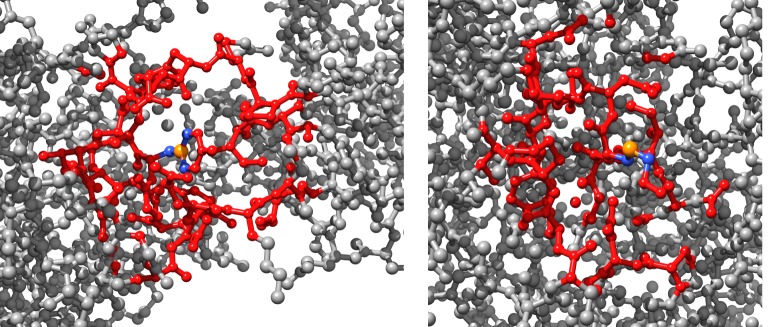
Examples of neighbourhoods. Central nodes, ‘owners’ of the neighbourhoods are coloured orange, their hard neighbours are blue and their soft neighbours are red.

**Figure 3.**
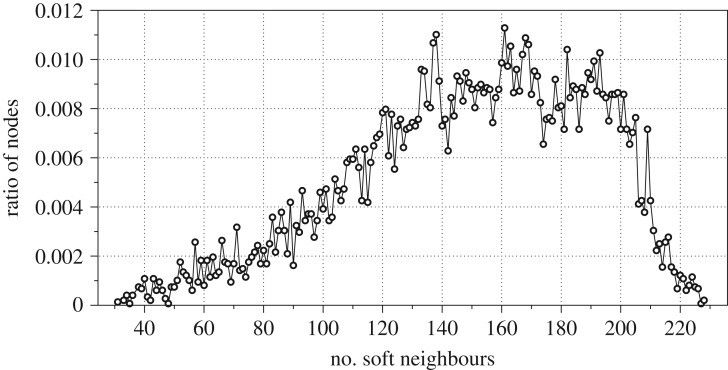
Distribution of a ratio of nodes versus numbers of their soft neighbours, *ρ* = 10.

The actin molecule is folded in the three-dimensional Euclidean space. Let *δ* be an average distance between two hard neighbours, for F-actin *δ* = 1.43 Å units. Let *w*(*p*) be the set of nodes of F that are at distance not exceed *ρ*, in the Euclidean space, from node *p*. We call them soft neighbours because their neighbourhood is determined by the three-dimensional structure of F-actin. Thus, each node *p* has two neighbourhoods: hard neighbourhood *u*(*p*) = {*s* ∈ **V**:(*ps*) ∈ **E**} (actin automata with hard neighbourhood were firstly proposed by us in [28]), and soft neighbourhood *w*(*p*) = {*s* ∈ **V**:*s*∉*u*(*p*) and *d*(*c*_*p*_, *c*_*s*_) ≤ *ρ*}, where *d*(*c*_*p*_, *c*_*s*_) is a distance between nodes *p* and *s* in three-dimensional Euclidean space and *c*_*s*_, *c*_*p*_ ∈ **C**. Interactions between a node and its hard neighbours takes place via atomic bounds and via the node and its soft neighbours via ionic currents. We have chosen *ρ* = 10 Å, which is seven times more than an average Euclidean distance 1.42 Å between two hard neighbours. Examples of neighbourhoods are shown in figure 2. The distribution of a number of soft neighbours versus a ratio of nodes with such number of soft neighbours is shown in figure 3; nearly half of the nodes (ratio 0.45) has from 133 to 185 neighbours. The ratio *γ*(*p*)^*t*^ is calculated as *γ*(*p*)^*t*^ = |*s* ∈ *u*(*p*):*s*^*t*^ = ⋆| + *μ* · |*s* ∈ *w*(*p*):*s*^*t*^ = ⋆|/|*u*(*p*)| + |*w*(*p*)|, where |**S**| is a number of elements in the set **S** and *μ* is a weight of soft neighbours; we used *μ* = 0.9 in experiments reported.

We consider two species of family F: semi-totalistic automaton $G=\langle V,\;E,\;C,\;\{⋆,\circ \},\;f^{G}\rangle$ and excitable automaton $E=\langle V,\;E,\;C,\;\{⋆,\circ ,•\},\;f^{E}\rangle$. The rules *f*^*G*^ and *f*^*E*^ are defined as follows:

2.1 $$p^{t+1}=f^{G}(p)=\left\{ \begin{array}{rl} ⋆, & \;\;\text{if }((p^{t}=\circ )\wedge (\theta_{\circ }^{\prime }\leq \gamma {\left(p\right)}^{t}\leq \theta_{\circ }^{\prime \prime }))\vee ((p^{t}=\;⋆)\wedge (\theta_{⋆}^{\prime }\leq \gamma {\left(p\right)}^{t}\leq \theta_{⋆}^{\prime \prime }))\\ \circ , & \;\;\text{otherwise } \end{array}\right.$$

2.2 $$p^{t+1}=f^{E}(p)=\left\{ \begin{array}{rl} ⋆, & \text{if }((p^{t}=\circ )\wedge (\theta_{\circ }^{\prime }\leq \gamma {\left(p\right)}^{t}\leq \theta_{\circ }^{\prime \prime }))\\ •, & \text{if }p^{t}=\circ \\ \circ , & \text{otherwise. } \end{array}\right.$$

We have chosen intervals [*θ*′_○_, *θ*′′_○_] = [*θ*′_⋆_, *θ*′′_⋆_] = [0.25, 0.375] for G and [*θ*′_○_, *θ*′′_○_] = [0.15, 0.25] for E because they support localized modes of excitation, i.e. a perturbation of the automata at a single site or a compact domain of several sites does not lead to an excitation spreading all over the actin chain. Localized excitations emerged at different input domains can interact with other and the results of their interactions in the output domains will represent values of a logical function computed.

**Figure 4.**
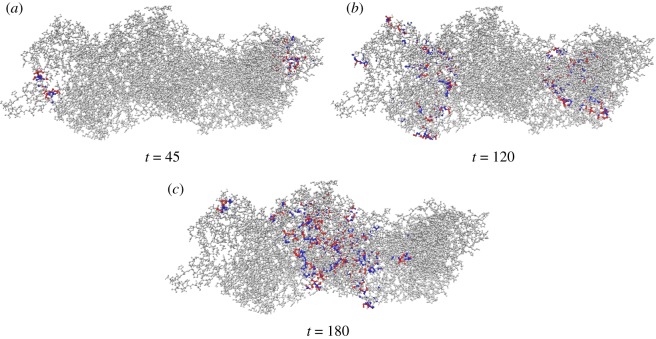
Annihilation of excitation wave-fronts in E for [*θ*′_○_, *θ*′′_○_] = [0.125, 1].

Automaton G is a Game of Life like automaton [29,30]. Speaking in the Game of Life lingo we can say that a dead node ○ becomes alive ⋆ if a ratio of live nodes in its neighbourhood lies inside interval [*θ*′_○_, *θ*′′_○_]; a live node ⋆ remains alive if a ratio of live nodes in its neighbourhood lies inside interval [*θ*′_⋆_, *θ*′′_⋆_]. Automaton E is a Greenberg–Hastings [31] like automaton: a resting node ○ excites if a ratio of excited nodes in its neighbourhood lies inside interval [*θ*′_○_, *θ*′′_○_]; and excited node ⋆ takes refractory state • in the next step of development, and a refractory • returns to resting state ○. Rules of Conway's Game of Life could be interpreted as equation (2.1) having perturbation intervals [*θ*′_○_, *θ*′′_○_] = [0.375, 0.375] (i.e. exactly value 0.375) and [*θ*′_⋆_, *θ*′′_⋆_] = [0.25, 0.375], rules of Greenberg–Hasting automata in terms of equation (2.2) having interval [*θ*′_○_, *θ*′′_○_] = [0.125, 1]. The exact intervals of perturbation for the Game of Life and the Greenberg–Hasting automata are proven to be not useful for mining functions. This is because G with the Game of Life interval does not show any sustainable dynamics of excitation, and E with Greenberg–Hasting interval exhibits ‘classical’ waves of excitation, where two colliding waves annihilate (figure 4).

The model was implemented in Processing. Data are analysed in Matlab. Patterns of excitation dynamics are visualized in Processing and Chimera.

## 3. Discovering functions

**Figure 5.**
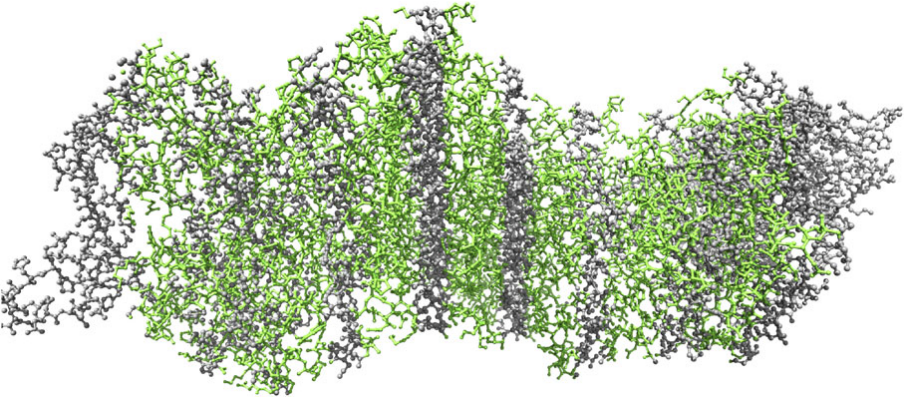
Nodes of I/O domains *D*_0_ … *D*_7_ are shown by green colour.

We encode Boolean values ‘0’ (False) and ‘1’ (True) in perturbations of selected domains ***D*** and extract a range of mappings ${\left\{0,\;1\right\}}^{m}\to {\left\{0,\;1\right\}}^{m}$, *m* ∈ ***N***, implementable by the actin filament automaton. Assume input and output tuples ***I*** ∈ {0, 1}^*m*^ and ***O*** ∈ {0, 1}^*m*^, *m* = 8, the actin automaton implements I→D→O. We implement computation on actin filament automaton as follows. Eight cylinders across the (*xy*)-plane with coordinates $D_{i}=\{p\in V:\;\text{abs}(p_{x}-k(i))<r_{s}\}$, 0 ≤ *i* < 8, *k*(*i*) = 15 · (*i* + 1), are assigned as input–output domains (figure 5). These are mapped onto Boolean inputs ***I*** = (*I*_0_, …, *I*_7_) and outputs **O** = (*O*_0_, …, *O*_7_) as follows: *I*_*z*_ = 1 if $\sum_{\;p^{0}\in D_{z}}>\kappa$, otherwise *I*_*z*_ = 0, and *O*_*z*_ = 1 if $\sum_{\;p^{\zeta }\in D_{z}}>\kappa$, otherwise *O*_*z*_ = 0; in the present paper we have chosen *κ* = 0 and *ζ* = 40.

Domains from **D** at time step *t* = 0 are excited with probability *p* determined by values of inputs **I**: if a node *p* belongs to **D**_i_ and *s*_*i*_ = 1 the node takes state ⋆ at the beginning of evolution, *p*^0^ = ⋆ with probability *p*. We read outputs after *ζ* = 40 steps of automaton evolution. As soon as 40 iterations occurred (*t* = 41), we measure states of nodes in the domains **D**_i_, *s*_*i*_ ∈ {0, 1}, and assign outputs depending on the excitation: *O*_*i*_ = 1 if |{*p* ∈ **D** : *p*^*t*^ = ⋆}| > *κ*, *κ* = 0. Stimulation runs for *h* trials (repeated simulation of automaton) with all possible configurations of ***I***, *h* = 100, where frequencies of outputs are calculated as *W*_*i*_ = *w*_*i*_ + *I*^*T*^_*i*_, 0 ≤ *i* < 8, where *T* is a trial number, *T* = 1, …, *h*. By the end of the experiments, we normalize **W** as *w*_*i*_ = *w*_*i*_/*h*, *h* is the number of trials.

**Figure 6.**
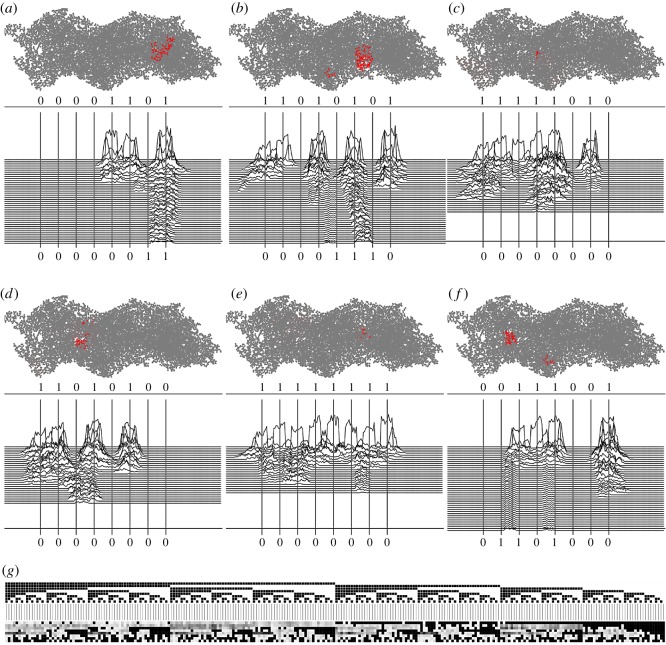
Discovering Boolean functions in automaton G. (*a*–*f*) Examples of excitation dynamics in automaton G, *θ*′_○_ = *θ*′_⋆_ = 0.25 and *θ*′′_○_ = *θ*′′_⋆_ = 0.375. Projection of actin filament on *z*-plane is shown in grey; projection of nodes being in state ⋆ by the moment of recording inputs are shown in red. Plots show values of activity, i.e. a number of nodes in state ⋆ along the *x*-coordinate. See videos of experiments at https://doi.org/10.5281/zenodo.1312141. (*g*) Visualization of register mapping implemented by automaton G.

Examples of perturbation dynamics of automaton G for various input sequences are shown in figure 6*a*–*f*. Example of a fragment of **W** obtained in 100 trials with automaton G is shown in table 1. Visualization of mapping S→W is presented in figure 6*g*. There, lexicograpically ordered elements of **S** are shown by black (‘1’) and white (‘0’) squares: top row from (0000000) on the right to (11111111) on the left. Corresponding elements of **W** are shown by gradations of grey 255 · *w*_*i*_. From **W**, we extract values of outputs **O** for various ranges of *γ* ∈ [0, 1] as follows: *O*_*i*_ = 1 if *w*_*i*_ > *γ*, and *O*_*i*_ = 0 otherwise.

**Table 1.** Fragment of experimentally obtained mapping **S** to **W** for automaton G.

(*I*_0_*I*_1_*I*_2_*I*_3_*I*_4_*I*_5_*I*_6_*I*_7_)	*w*_0_	*w*_1_	*w*_2_	*w*_3_	*w*_4_	*w*_5_	*w*_6_	*w*_7_
1011100	0	0	0.01	0.01	0.14	0.03	0.01	0
1011101	0.01	0.03	0.01	0.01	0.2	0.03	0.01	0.03
1011110	0	0	0	0	0.14	0.02	0.01	0.01
1011111	0	0.01	0.01	0.02	0.25	0.04	0.02	0.02
1100000	0	0.04	0.04	0	0	0	0	0
1100001	0	0.02	0.02	0	0	0	0	0.01
1100010	0	0.03	0.05	0	0	0.01	0.02	0
1100011	0	0.05	0.03	0	0	0	0	0.02
1100100	0	0.06	0.04	0	0	0.02	0	0
1100101	0.01	0.06	0.04	0	0	0.04	0.03	0.02

**Figure 7.**
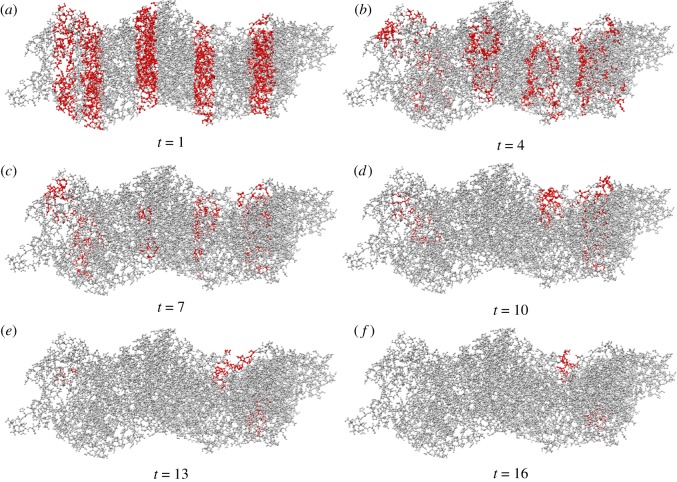
Snapshots of excitation dynamics of automaton G in response to the input 11010101. See videos of experiments at https://doi.org/10.5281/zenodo.1312141.

**Figure 8.**
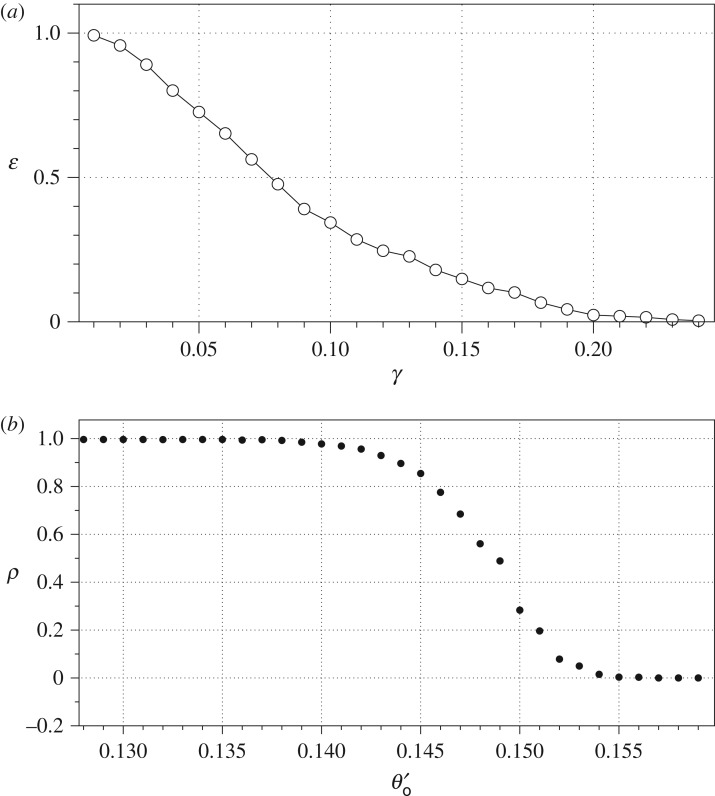
(*a*) Ratio *ɛ* of transitions where at least one entry in ***W*** exceeds *γ*. (*b*) Dependence of the ratio *ρ* of outputs in state 1 to an overall number of outputs of the lower threshold of excitation *θ*′_○_; upper threshold *θ*′_○_ = 0.25 was kept constant.

Boolean functions, in the form *O*_*i*_ = *f*(*I*_0_ … *I*_7_), realizable by automata G and E are listed in table 2. In automaton G a ratio *ε* of I/O transitions where at least one element of **W** exceeds *γ* shows quadratic decrease with increase of *γ* (figures 7 and 8*a*); the same applies to automaton E. This reflects both a decrease in a number of functions realizable on output domains and a decrease of the functions complexity in terms of the arguments. A number of functions implementable in F polynomially decrease with increase of *θ*′_○_ (figure 8*b*).

**Table 2.** Functions implemented by (*a*) G automaton, *θ*′_○_ = *θ*′_⋆_ = 0.25 and *θ*′′_○_ = *θ*′′_⋆_ = 0.375, and (*b*) E automaton, *θ*′_○_ = 0.15 and *θ*′′_○_ = 0.25. for various values of reliability threshold *γ*.

*γ*	functions
(*a*)	
0.15	$O_{1}=I_{0}\cdot \bar{I_{1}}\cdot I_{2}\cdot \bar{I_{3}}\cdot I_{4}\cdot I_{5}\cdot I_{6}\cdot I_{7}$;
	$O_{2}=\bar{I_{0}}\cdot \bar{I_{1}}\cdot I_{2}\cdot I_{3}\cdot I_{7}\cdot (I_{4}\cdot I_{5}\cdot \bar{I_{6}}+I_{4}\cdot \bar{I_{5}}\cdot I_{6}+\bar{I_{4}}\cdot I_{5}\cdot I_{6})$;
	$O_{4}=\bar{I_{0}}\cdot \bar{I_{1}}\cdot \bar{I_{2}}\cdot I_{3}\cdot I_{4}\cdot (\bar{I_{5}}\cdot \bar{I_{7}}+I_{6}\cdot I_{7}+I_{5}\cdot I_{6}\cdot \bar{I_{7}})$
0.2	$O_{4}=I_{3}\cdot I_{4}\cdot (\bar{I_{0}}\cdot I_{1}\cdot \bar{I_{2}}\cdot I_{5}\cdot I_{7}+\bar{I_{0}}\cdot \bar{I_{2}}\cdot \bar{I_{5}}\cdot \bar{I_{6}}\cdot \bar{I_{7}}+I_{0}\cdot \bar{I_{1}}\cdot \bar{I_{2}}\cdot \bar{I_{5}}\cdot I_{6}+\bar{I_{0}}\cdot I_{1}\cdot I_{2}\cdot \bar{I_{5}}\cdot I_{6}\cdot$
	$\bar{I_{7}}+I_{0}\cdot I_{1}\cdot I_{2}\cdot \bar{I_{5}}\cdot I_{6}\cdot I_{7}+I_{0}\cdot \bar{I_{1}}\cdot \bar{I_{2}}\cdot \bar{I_{5}}\cdot \bar{I_{6}}\cdot I_{7}+\bar{I_{0}}\cdot \bar{I_{1}}\cdot \bar{I_{2}}\cdot I_{5}\cdot I_{6}\cdot \bar{I_{7}}+\bar{I_{0}}\cdot \bar{I_{1}}\cdot \bar{I_{2}}\cdot \bar{I_{5}}\cdot I_{6}\cdot I_{7})$
0.22	$O_{4}=\bar{I_{2}}\cdot I_{3}\cdot I_{4}\cdot (\bar{I_{0}}\cdot I_{1}\cdot I_{5}\cdot I_{6}\cdot I_{7}+\bar{I_{0}}\cdot \bar{I_{1}}\cdot \bar{I_{5}}\cdot \bar{I_{6}}\cdot \bar{I_{7}}+I_{0}\cdot \bar{I_{1}}\cdot \bar{I_{5}}\cdot \bar{I_{6}}\cdot I_{7}+\bar{I_{0}}\cdot \bar{I_{1}}\cdot I_{5}\cdot I_{6}\cdot$
	$\bar{I_{7}}+\bar{I_{0}}\cdot \bar{I_{1}}\cdot \bar{I_{5}}\cdot I_{6}\cdot I_{7})$
0.23	$O_{4}=\bar{I_{0}}\cdot \bar{I_{2}}\cdot I_{3}\cdot I_{4}\cdot (\bar{I_{1}}\cdot I_{5}\cdot I_{6}\cdot \bar{I_{7}}+\bar{I_{1}}\cdot \bar{I_{5}}\cdot I_{6}\cdot I_{7}+I_{1}\cdot I_{5}\cdot I_{6}\cdot I_{7}+\bar{I_{1}}\cdot \bar{I_{5}}\cdot \bar{I_{6}}\cdot \bar{I_{7}})$
0.24	$O_{4}=\bar{I_{0}}\cdot \bar{I_{2}}\cdot I_{3}\cdot I_{4}\cdot I_{6}\cdot I_{7}\cdot (\bar{I_{1}}\cdot \bar{I_{5}}+I_{1}\cdot I_{5})$
0.25	$O_{4}=\bar{I_{0}}\cdot I_{1}\cdot \bar{I_{2}}\cdot I_{3}\cdot I_{4}\cdot I_{5}\cdot I_{6}\cdot I_{7}$
(*b*)	
0.7	$O_{0}=\bar{I_{0}}\cdot I_{1}\cdot \bar{I_{2}}\cdot I_{7}\cdot (\bar{I_{3}}\cdot \bar{I_{4}}\cdot I_{5}\cdot \bar{I_{6}}+I_{3}\cdot I_{4}\cdot \bar{I_{5}}\cdot I_{6})$
	$O_{1}=I_{0}\cdot \bar{I_{1}}\cdot I_{2}\cdot \bar{I_{3}}\cdot I_{4}+\bar{I_{0}}\cdot I_{1}\cdot \bar{I_{2}}\cdot I_{3}\cdot I_{4}\cdot \bar{I_{5}}\cdot I_{7}+\bar{I_{0}}\cdot I_{1}\cdot \bar{I_{2}}\cdot I_{3}\cdot \bar{I_{4}}\cdot I_{5}\cdot \bar{I_{6}}+\bar{I_{0}}\cdot I_{1}\cdot \bar{I_{2}}\cdot I_{3}\cdot \bar{I_{4}}\cdot I_{6}\cdot$
	$\bar{I_{7}}+\bar{I_{0}}\cdot I_{1}\cdot I_{2}\cdot \bar{I_{3}}\cdot I_{4}\cdot \bar{I_{5}}\cdot \bar{I_{6}}\cdot I_{7}+\bar{I_{0}}\cdot I_{1}\cdot I_{2}\cdot \bar{I_{3}}\cdot I_{4}\cdot I_{5}\cdot I_{6}\cdot I_{7}+I_{0}\cdot \bar{I_{1}}\cdot I_{2}\cdot I_{3}\cdot \bar{I_{4}}\cdot I_{5}\cdot \bar{I_{6}}\cdot I_{7}+\bar{I_{0}}\cdot$
	$I_{1}\cdot \bar{I_{2}}\cdot I_{3}\cdot \bar{I_{4}}\cdot I_{5}\cdot I_{6}\cdot I_{7}$
	$O_{2}=I_{0}\cdot \bar{I_{1}}\cdot I_{2}\cdot \bar{I_{3}}\cdot I_{4}+\bar{I_{0}}\cdot I_{1}\cdot I_{2}\cdot \bar{I_{3}}\cdot I_{4}\cdot \bar{I_{5}}+I_{0}\cdot \bar{I_{1}}\cdot I_{2}\cdot I_{3}\cdot \bar{I_{4}}\cdot I_{5}\cdot \bar{I_{7}}+\bar{I_{0}}\cdot I_{1}\cdot \bar{I_{2}}\cdot$
	$I_{3}\cdot \bar{I_{4}}\cdot I_{6}\cdot \bar{I_{7}}+\bar{I_{0}}\cdot I_{1}\cdot \bar{I_{2}}\cdot I_{3}\cdot \bar{I_{4}}\cdot \bar{I_{6}}\cdot I_{7}+\bar{I_{0}}\cdot I_{1}\cdot I_{3}\cdot \bar{I_{4}}\cdot I_{5}\cdot \bar{I_{6}}\cdot \bar{I_{7}}+\bar{I_{0}}\cdot I_{1}\cdot \bar{I_{2}}\cdot I_{3}\cdot I_{4}\cdot \bar{I_{5}}\cdot$
	$\bar{I_{6}}\cdot I_{7}+I_{0}\cdot I_{1}\cdot \bar{I_{2}}\cdot I_{3}\cdot$ $\bar{I_{4}}\cdot I_{5}\cdot \bar{I_{6}}\cdot I_{7}+I_{0}\cdot \bar{I_{1}}\cdot I_{2}\cdot I_{3}\cdot \bar{I_{4}}\cdot I_{5}\cdot \bar{I_{6}}\cdot I_{7}+\bar{I_{0}}\cdot I_{1}\cdot I_{2}\cdot I_{3}\cdot \bar{I_{4}}\cdot$
	$I_{5}\cdot \bar{I_{6}}\cdot I_{7}+\bar{I_{0}}\cdot I_{1}\cdot I_{2}\cdot \bar{I_{3}}\cdot I_{4}\cdot I_{5}\cdot I_{6}\cdot \bar{I_{7}}+\bar{I_{0}}\cdot$ $I_{1}\cdot \bar{I_{2}}\cdot I_{3}\cdot \bar{I_{4}}\cdot I_{5}\cdot I_{6}\cdot I_{7}$
	$O_{3}=I_{2}\cdot \bar{I_{3}}\cdot I_{4}\cdot \bar{I_{5}}\cdot \bar{I_{6}}\cdot I_{7}+I_{0}\cdot I_{3}\cdot \bar{I_{4}}\cdot I_{5}\cdot \bar{I_{6}}\cdot I_{7}+\bar{I_{0}}\cdot I_{1}\cdot I_{2}\cdot I_{3}\cdot \bar{I_{4}}\cdot I_{5}\cdot \bar{I_{7}}+\bar{I_{0}}\cdot I_{1}\cdot I_{3}\cdot \bar{I_{4}}\cdot I_{5}\cdot$
	$\bar{I_{6}}\cdot I_{7}+\bar{I_{1}}\cdot I_{2}\cdot I_{3}\cdot \bar{I_{4}}\cdot$ $I_{5}\cdot I_{6}\cdot \bar{I_{7}}+I_{0}\cdot \bar{I_{1}}\cdot I_{2}\cdot \bar{I_{3}}\cdot I_{4}\cdot \bar{I_{5}}\cdot \bar{I_{7}}+\bar{I_{0}}\cdot I_{1}\cdot I_{2}\cdot \bar{I_{3}}\cdot I_{4}\cdot$
	$\bar{I_{5}}\cdot \bar{I_{7}}+\bar{I_{0}}\cdot \bar{I_{2}}\cdot I_{3}\cdot \bar{I_{4}}\cdot I_{5}\cdot I_{6}\cdot \bar{I_{7}}+I_{1}\cdot \bar{I_{2}}\cdot I_{3}\cdot \bar{I_{4}}\cdot I_{5}\cdot \bar{I_{6}}\cdot \bar{I_{7}}+I_{0}\cdot I_{1}\cdot \bar{I_{2}}\cdot I_{3}\cdot \bar{I_{4}}\cdot I_{5}\cdot I_{6}+I_{0}\cdot \bar{I_{1}}\cdot I_{2}\cdot$
	$I_{3}\cdot \bar{I_{4}}\cdot I_{5}\cdot$ $\bar{I_{6}}\cdot \bar{I_{7}}+\bar{I_{0}}\cdot \bar{I_{1}}\cdot I_{2}\cdot I_{3}\cdot \bar{I_{4}}\cdot I_{5}\cdot \bar{I_{6}}\cdot I_{7}+I_{0}\cdot I_{1}\cdot I_{2}\cdot \bar{I_{3}}\cdot I_{4}\cdot \bar{I_{5}}\cdot I_{6}\cdot \bar{I_{7}}+I_{0}\cdot \bar{I_{1}}\cdot I_{2}\cdot \bar{I_{3}}\cdot I_{4}\cdot \bar{I_{5}}\cdot$
	$I_{6}\cdot I_{7}+\bar{I_{0}}\cdot I_{1}\cdot I_{2}$ $\bar{I_{3}}\cdot I_{4}\cdot \bar{I_{5}}\cdot I_{6}\cdot I_{7}+\bar{I_{0}}\cdot \bar{I_{1}}\cdot I_{2}\cdot \bar{I_{3}}\cdot I_{4}\cdot \bar{I_{5}}\cdot \bar{I_{6}}\cdot \bar{I_{7}}$
	functions realized on outputs *O*_4_ to *O*_7_ are not shown.
0.8	$O_{2}=I_{3}\cdot \bar{I_{4}}\cdot I_{5}\cdot I_{7}\cdot (\bar{I_{0}}\cdot I_{1}\cdot \bar{I_{2}}+I_{0}\cdot \bar{I_{1}}\cdot I_{2}\cdot \bar{I_{6}})$
	$O_{3}=\bar{I_{6}}\cdot (I_{0}\cdot \bar{I_{1}}\cdot I_{3}\cdot \bar{I_{4}}\cdot I_{5}\cdot I_{7}+\bar{I_{0}}\cdot I_{1}\cdot I_{3}\cdot \bar{I_{4}}\cdot I_{5}\cdot$
	$I_{7}+I_{0}\cdot \bar{I_{1}}\cdot I_{2}\cdot I_{3}\cdot \bar{I_{4}}\cdot I_{5}\cdot \bar{I_{7}}+I_{0}\cdot \bar{I_{1}}\cdot I_{2}\cdot \bar{I_{3}}\cdot I_{4}\cdot$
	$\bar{I_{5}}\cdot I_{7}+I_{0}\cdot I_{1}\cdot \bar{I_{2}}\cdot I_{3}\cdot \bar{I_{4}}\cdot I_{5}\cdot I_{7})$
	$O_{4}=\bar{I_{6}}\cdot (I_{2}\cdot I_{3}\cdot \bar{I_{4}}\cdot I_{5}\cdot I_{7}+\bar{I_{1}}\cdot I_{2}\cdot \bar{I_{3}}\cdot I_{4}\cdot \bar{I_{5}}\cdot I_{7}+I_{0}\cdot I_{1}\cdot \bar{I_{2}}\cdot I_{3}\cdot \bar{I_{4}}\cdot I_{5}+I_{0}\cdot \bar{I_{1}}\cdot \bar{I_{2}}\cdot I_{3}\cdot \bar{I_{4}}\cdot I_{5}\cdot I_{7})$
	$O_{5}=\bar{I_{2}}\cdot I_{3}\cdot \bar{I_{4}}\cdot I_{5}\cdot \bar{I_{6}}\cdot I_{7}\cdot (I_{0}+\bar{I_{1}})$
0.9	$O_{5}=\bar{I_{1}}\cdot I_{3}\cdot \bar{I_{4}}\cdot I_{5}\cdot \bar{I_{6}}\cdot I_{7}\cdot (I_{0}+\bar{I_{2}})$

## 4. Discussion

We demonstrated an implementation of logical functions on automaton models of actin filaments. The approach was inspired by the ‘evolution in materio’ framework [32–34] on implementing computation without knowing the exact physical structure of a computing substrate. Propagating patterns in the Game of Life like automaton G can be seen as the discrete analogies of vibration excitation [35–38]. The dynamics of Greenberg–Hasting excitable automaton E is a finite-state machine analogue of the ionic waves, theoretical models of which are well studied in a context of tubulin microtubules and actin filaments [7,10,39–41]. How feasible is the approach? So far there are no experimental data on vibration modes of a single strand, or even a bundle of actin filaments or tubulin tubes, of a cytoskeleton polymer [42]. Outputs of the actin filament processors can be measured using controlled light waves and pulse trains [43–46]. There are ways to measure a vibration of a cell membrane, as demonstrated in [47]. The vibration of the membrane might reflect vibrations of cytoskeleton networks attached to the membrane [48], however it shows a cumulative effect of vibration of a cytoskeleton network. Owing to the polarity of actin units, vibration modes are manifested in electromagnetic perturbation which could be measured when existing experimental techniques are perfected [37,38].

## Footnotes

1 PDB file can be downloaded here https://www.rcsb.org/structure/3J8I.

## Data accessibility

The data supporting the findings of this study are available at https://doi.org/10.5281/zenodo.1312141.
